# Missing driver in the Sun–Earth connection from energetic electron precipitation impacts mesospheric ozone

**DOI:** 10.1038/ncomms6197

**Published:** 2014-10-14

**Authors:** M. E. Andersson, P. T. Verronen, C. J. Rodger, M. A. Clilverd, A. Seppälä

**Affiliations:** 1 Earth Observation, Finnish Meteorological Institute, PO Box 503 (Erik Palménin aukio 1), Helsinki FI-00101, Finland; 2 Department of Physics, University of Otago, PO Box 56, Dunedin 9016, New Zealand; 3 British Antarctic Survey (NERC), High Cross, Madingley Road, Cambridge CB3 0ET, UK

## Abstract

Energetic electron precipitation (EEP) from the Earth’s outer radiation belt continuously affects the chemical composition of the polar mesosphere. EEP can contribute to catalytic ozone loss in the mesosphere through ionization and enhanced production of odd hydrogen. However, the long-term mesospheric ozone variability caused by EEP has not been quantified or confirmed to date. Here we show, using observations from three different satellite instruments, that EEP events strongly affect ozone at 60–80 km, leading to extremely large (up to 90%) short-term ozone depletion. This impact is comparable to that of large, but much less frequent, solar proton events. On solar cycle timescales, we find that EEP causes ozone variations of up to 34% at 70–80 km. With such a magnitude, it is reasonable to suspect that EEP could be an important part of solar influence on the atmosphere and climate system.

## Abstract (web summary)

Energetic electron precipitation (EEP) from the Earth's outer radiation belt can lead to ozone loss in the mesosphere, yet long-term variability has not been quantified. Here, the authors present satellite observations and show that on solar cycle timescales EEP causes ozone to vary by up to 34%.

## Graphical abstract

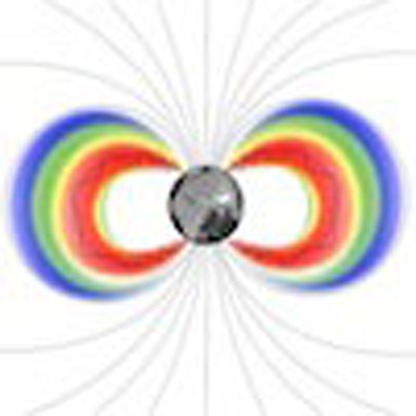

Energetic electron precipitation (EEP) from the Earth’s outer radiation belt continuously affects the chemical composition of the mesosphere across the geomagnetic latitudes 55–65°. At altitudes below ~80 km, EEP leads to odd hydrogen (HO_*x*_) enhancement following ionization and ion chemical reactions^1^, which is expected to contribute to the ozone balance in the mesosphere. A recent study considering the 2004–2009 period concluded that EEP was significantly affecting mesospheric HO_*x*_ around 35% of the time^2^. A set of case studies has demonstrated that EEP-HO_*x*_ is expected to have a short-term effect on mesospheric ozone through well-known catalytic reaction chains^3^. The largest effects of EEP on HO_*x*_ have been reported at 70–80 km, caused by electrons with energies between 100 and 300 keV. The EEP effect is most significant during and following geomagnetic storms, where dynamic processes inside the radiation belts accelerate electrons to high energies.

Here we show, using ozone observations from three different satellite instruments, that EEP events very strongly affect ozone at altitudes between 60 and 80 km. The EEP leads to an extremely large (up to 90%) short-term (days) ozone depletion in the atmosphere. The magnitude of these short-term effects is comparable to those caused by large but much less frequent solar proton events^4^,^5^. On solar cycle scales, we find that EEP causes significant ozone variations of up to 34% at 70–80 km. As ozone is important to atmospheric heating and cooling rates, this level of ozone variation could significantly affect the local mesospheric temperature balance^6^. Our results emphasize the importance of the EEP effect on mesospheric ozone and significantly improve our understanding of the impacts of the energetic particles on the atmosphere.

## Results

### EEP in 2002–2012

**Figure 1:**
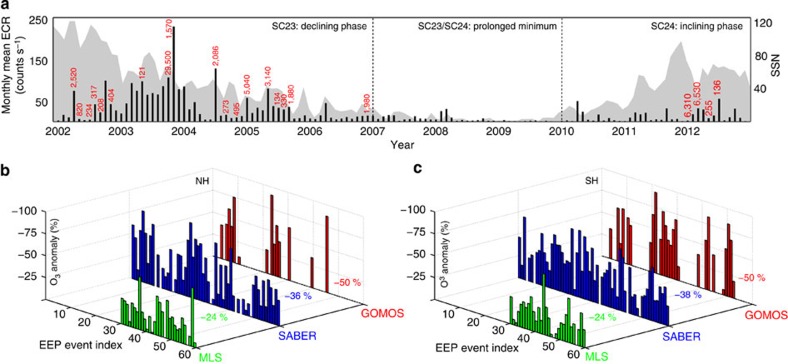
Signature of EEP in observed mesospheric ozone. (**a**) Monthly mean ECRs (black bars), maximum proton flux >10 MeV (red numbers) in proton flux units (1 pfu=1 p cm^−2^ sr^−1^ s^−1^) and sunspot number (SSN, grey area) between 2002 and 2012. (**b**,**c**) Maximum O_3_ loss (%) at altitudes between 70 and 78 km in the Northern hemisphere (**b**) and Southern hemisphere (**c**) during 60 EEP events, with daily ECR >150 (counts s^−1^). Numbers: the average O_3_ loss (%) for each set of available satellite measurements (MLS, SABER and GOMOS).

Solar cycle 23 (SC23) was one of the longest cycles since 1847 and exhibited large variation in solar (UV radiation) and geomagnetic activity (solar storms, energetic particle precipitation). In 2003, during the declining phase of SC23, the majority of the days were geomagnetically disturbed. In contrast, the deep solar minimum that occurred in 2009 showed the lowest activity since the beginning of the Twentieth century. The current solar cycle (SC24) is so far the weakest cycle in the last 100 years. For this period, EEP events were strongest and most frequent during the transition between SC23 maximum and the following minimum (Fig. 1a). Almost 75% of all major EEP events (major=daily mean electron precipitation count rate exceeding 150 counts s^−1^) in the 2002–2012 period occurred between 2003 and 2006. The occurrence of solar proton events (SPEs) peaked during high solar activity (red numbers in Fig. 1a).

### Overview of ozone-depleting events

We now consider the 60 major EEP events that occurred between 2002 and 2012. For these, the satellite measurements show EEP-induced ozone loss occurring consistently in both hemispheres (Fig. 1b,c). The maximum relative ozone depletion during EEP events occurs at altitudes between 70 and 78 km and varies from 5 to 90% across the events. The average response is 50, 37 and 24% for the Global Ozone Monitoring by Occultation of Stars (GOMOS), Sounding of the Atmosphere using Broadband Emission Radiometry (SABER) and Microwave Limb Sounder (MLS) ozone observations, respectively. The differences between the average responses is partially because of the different vertical resolutions of the observations but is also connected to data availability for some events. For example, the MLS data do not cover the period 2002–2004 that contained multiple extremely strong and long-lasting EEP events.

### Short-term ozone depletion

**Figure 2:**
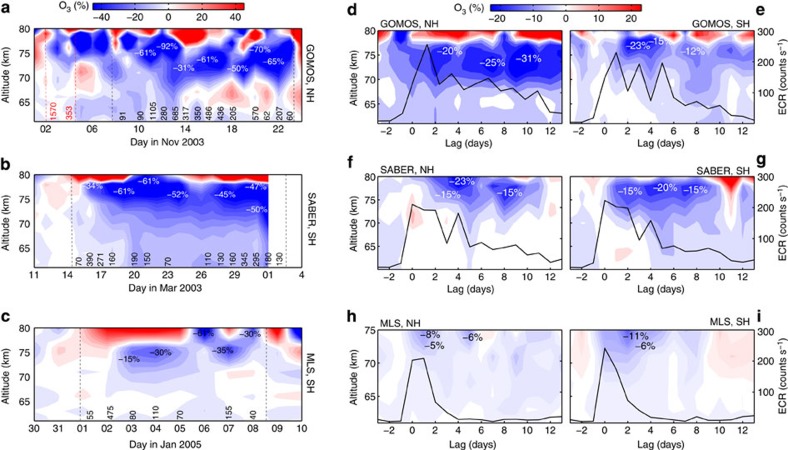
Magnitude of the short-term EEP effects on mesospheric ozone. (**a**–**c**) O_3_ anomalies (%) for selected EEP events in the Northern hemisphere and in the Southern hemisphere derived from GOMOS (**a**), SABER (**b**) and MLS (**c**) observations. Black dashed lines: EEP event start end end; red dashed lines: SPE event start end end; black numbers: daily mean ECRs; red numbers: >10 MeV pfu. (**d**–**i**) Superposed epoch analysis for EEP events with daily ECR >150 (counts s^−1^) showing ozone anomalies (%) and ECR (black lines) in the Northern hemisphere (**d**,**f**,**h**) and in the Southern hemisphere (**e**,**g**,**i**). White numbers: O_3_ loss at different altitudes.

The response of mesospheric ozone to EEP is immediate; however, the magnitude and duration of the depletion can differ depending on both the characteristics of the event as well as the season (Fig. 2a–c). During strong EEP events lasting more than 5 days, for example, 03/2003, 11/2003 and 01/2005, significant ozone depletion of up to 90% is seen at altitudes 75–80 km, with the impact reaching down to 60 km altitude. Over the 60- to 80-km altitude range, these events are comparable to the effects of large SPEs. Shorter EEP events (1–5 days, Supplementary Fig. 1a,b) usually affect altitudes between 65 and 80 km with maximum O_3_ decreases of 70%. The effect of EEP is typically more pronounced during the wintertime (Fig. 2a, Supplementary Fig. 1b), as the EEP-HO_*x*_ production is then relatively larger when compared with the background HO_*x*_ production by photodissociation of water vapour. Of the 60 EEP events, the one on 9–23 November 2003 caused the strongest ozone depletion (Fig. 2a). The event lasted 15 days, with major forcing on 10 of those days and occurred right after Halloween 2003 SPE event. This EEP event had ozone depleted by maximum of 92%, a day after the strongest EEP forcing on 11 November. Although in principle the ozone depletion caused by the Halloween SPE could influence the EEP event period, the GOMOS observations (Fig. 2a) as well as observations at higher latitudes from the MIPAS and SCIAMACHY instruments^7^,^8^,^9^ show that, in agreement with modelling, the mesospheric ozone recovered from the effects of the SPE event by 7–8 November, before the strong EEP forcing is observed.

### Superposed epoch analysis

To assess the sensitivity and robustness of our results, we carried out a superposed epoch analysis of the 60 largest EEP events (Fig. 2d–i). All SPE periods that could possibly affect the results were excluded from the analysis. The ozone depletion coincides closely with EEP increases and can last from 3 to 10 days, depending on the EEP duration. As MLS does not cover years 2003–2004, during which many strong and long-lasting EEP events occurred, the average O_3_ loss is weaker than in GOMOS and SABER data (Fig. 2h–i). The maximum loss of ozone occurs between 70 and 78 km altitude with magnitudes varying from 10 to 30%, depending on the number and strength of EEP events, instrument resolution and atmospheric conditions. A similar superposed epoch analysis for a randomly selected data set (Supplementary Fig. 2a–f) shows no negative response in ozone. The increasing trend in percentage difference in O_3_ in the random epoch analysis is caused by a seasonal bias in the observation data sets. This is particularly evident for GOMOS that generally has poorer coverage during summer periods. The superposed EEP events shown in Fig. 2 include this underlying trend effect but still show decreases in O_3_ percentage change nevertheless. The average ozone loss because of EEP (Fig. 2d–i) is clearly larger than the 95% confidence range for the random data set (Supplementary Fig. 2g–i). Finally, to address the seasonal variability, we carried out superposed epoch analysis separately for three different seasons: winter, summer and spring/autumn (Supplementary Fig. 3). The results confirm that, for the same EEP forcing, the ozone loss during the winter period is typically more pronounced, for example, stretching over a wider altitude range, than in the summer and autumn/spring seasons.

### Long-term ozone variability

**Figure 3:**
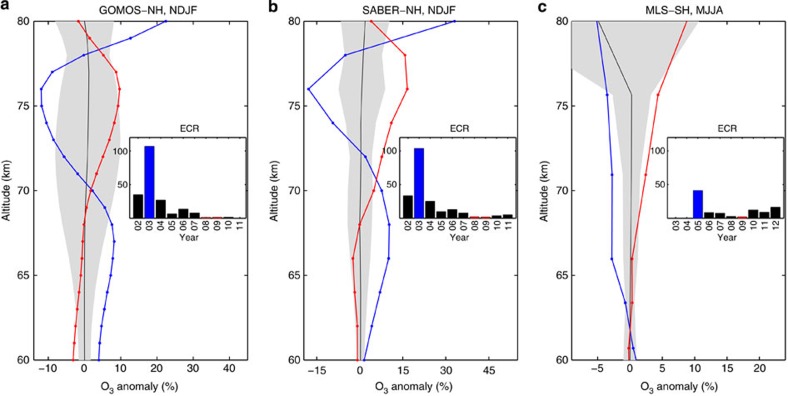
Magnitude of the long-term EEP effects on mesospheric ozone. (**a**–**c**) Ozone anomalies (%) of deseasonalized daily means, averaged over the winter time. (**a**) November to February in the Northern hemisphere from GOMOS showing years 2003 (blue line) and 2008–2009 (red line). (**b**) November to February in the Northern hemisphere from SABER showing years 2003 (blue line) and 2008–2009 (red line). (**c**) May to August in the Southern hemisphere from MLS showing years 2005 (blue line) and 2009 (red line). Black lines: winter time climatology from 2002 to 2012; grey area: 95% confidence range of the climatological mean. Subplots: winter time average ECRs between 2002 and 2012.

Although the duration of the forcing for individual EEP events is only a few days, the high frequency of the events during active years (Fig. 1a) is enough to cause variability in mesospheric ozone on solar cycle timescales (Fig. 3a–c). Determining EEP-related ozone anomaly as a function of year or solar cycle is not straight forward because the temporal distribution of EEP events does not smoothly vary across the solar cycle. For example, the majority of the strong EEP events were observed during the declining phase of SC23, with a peak in year 2003 (Fig. 1). Instead, we can look at the EEP impact by contrasting periods of maximum and minimum EEP activity, which is then an indication of the maximum variability during the solar cycle. On the basis of the strength and frequency of the EEP (Fig. 3a,b, Subplots), for GOMOS and SABER we selected wintertime 2003 and 2008–2009 as maximum and minimum EEP periods, respectively. For MLS data, because they do not cover 2003, we selected year 2005 to represent the EEP maximum (Fig. 3c, Subplot). Before the analysis, we carefully removed SPE-influenced periods from all data sets. For example, in November 2003 (Fig. 2a) we excluded days 1–8 which, according to previously published satellite observations of ozone^7^,^8^,^9^, were affected by the Halloween 2003 SPE. The wintertime ozone values are much smaller during the EEP maximum than during the EEP minimum. The largest differences, ~21% for GOMOS (Fig. 3a) and 34% for SABER (Fig. 3b), are observed at the altitudes of 70–80 km that are known to be most strongly affected by EEP. For MLS, the difference between years 2005 and 2009 is smaller (~9%), which is consistent with weaker forcing in 2005 compared with 2003 (Fig. 3, Subplots). Note that the ozone anomalies during the EEP maximum and minimum years are outside the 95% confidence range of the climatological mean from 2002 to 2012 (Fig. 3).

## Discussion

In recent years, the atmospheric effects of energetic particle precipitation (=EEP+SPE) have received a considerable amount of scientific attention. Most studies have concentrated on the so-called indirect particle precipitation effect caused by the production of odd nitrogen (NO_*x*_) in the polar upper atmosphere, its subsequent transport to lower altitudes inside the wintertime polar vortex, depletion of ozone in the stratosphere and effects on the radiative balance of the middle atmosphere^10^,^11^,^12^,^13^. These effects may further couple to atmospheric dynamics and propagate downwards by changing polar winds and atmospheric wave propagation through wave—mean flow interaction^14^,^15^,^16^. Several studies have suggested links between the EPP indirect effect on ozone and regional wintertime tropospheric climate variability^17^,^18^,^19^,^20^.

Our results show that the direct, HO_*x*_-driven effect of EEP is causing significant, previously unaccounted for, ozone variability in the mesosphere that are observable on solar cycle timescales. Although these effects from EEP-HO_*x*_ have not been considered in atmospheric and climate models to date, dynamical changes in the mesosphere and stratosphere have been reported as a result of SPEs and the indirect EEP impact on ozone^19^,^21^.

Considering the magnitude of the direct ozone effect, tens of percent in wintertime polar regions, it is reasonable to suspect that EEP could be an important contributor to the Sun-climate connection on solar cycle timescales. For comparison, the 11-year variability in UV radiation has a less than 10% effect on annual mean mesospheric ozone at mid-to-low latitude^22^,^23^,^24^. Thus, more research should be directed towards better understanding the potential further effects from EEP and its role in the overall Solar influence on climate. Currently, in most high-top climate models the solar input does not include EEP and it is completely missing from low-top models.

## Methods

### Satellite measurements

Measurements from GOMOS/Envisat, SABER/TIMED and MLS/Aura are used to estimate mesospheric ozone loss because of energetic electron precipitation. We use zonally averaged daily mean ozone profiles. Measurements from Medium-Energy Proton and Electron Detector (MEPED/POES) are used to estimate daily and monthly mean electron count rates (ECRs) and identify EEP events. Characteristics of these data sets are given below and in Supplementary Table 1. The anomalies in Figs 1b and 2 are calculated on a daily timescale with respect to a 7-day average before the EEPevent. The starting point for each event is defined as the first day of an EEP event with geomagnetic Ap index exceeding 20.

### GOMOS

The GOMOS (v6) O_3_ stellar occultation measurements^25^ used here were made at geomagnetic latitudes 55–65° N/S using stars with temperature >6,000 K. Data were selected, requiring the solar zenith angle (SZA) at the tangent point and the satellite point to be >95° and >90°, respectively. By selecting this SZA limit, we have increased number of profiles selected but include some observations under twilight conditions. The estimated error is of ~5% at altitudes between 70 and 80 km.

### SABER

The SABER (v1.07) O_3_ measurements^26^,^27^ used here were derived from the infrared emission observations at 9.6 μ. We have used measurements at geomagnetic latitudes 55–65° N/S with SZA>95°. The accuracy of the ozone measurements is of the order of 10% in the lower mesosphere region, with a positive bias increasing with altitude.

### MLS

The MLS (v3.3) O_3_ observations^28^,^29^ used here were recorded in the geomagnetic latitudinal range 55–65° N/S and with SZA>95°. The accuracy of the O_3_ measurements is of the order of 35%. Although the recommended upper altitude limit for MLS O_3_ scientific observations is ~75 km, we extended it up to 80 km to provide qualitative estimation of the changes caused by EEP.

### MEPED

Data used here were from the 0° detector pointing radially outwards along the Earth–satellite direction^30^,^31^. We utilize data from L shells spanning 3.0–5.5, equivalent to the geomagnetic latitudes of 55–65° and the locations of the inner and mid parts of the outer radiation belt. The precipitating ECRs measurements are considered the same way as in previous studies using the same data^2^. The count rates of the >300-keV energy channel are subtracted from those of the >100-keV channel to get an estimate of the flux of precipitating electrons that will deposit the majority of their energy into the atmosphere at altitudes of 70–80 km.

### Solar proton events

To identify and exclude the SPE events we use the list of SPEs and their magnitudes from NOAA Space Weather Prediction Center ( http://www.swpc.noaa.gov/ftpdir/indices/SPE.txt, accessed in July 2013).

### Superposed epoch analysis

Superposed epoch analysis is used to test the significance of ozone loss because of energetic electron precipitation. The analysis was carried out for three data sets: (1) identified EEP events where the daily average is ECR >150 (counts s^−1^) for at least 1 day of the event period, (2) randomly selected data and (3) three different seasons. For data set 1 (Fig. 2d–i), ozone anomalies during selected events are superposed on each other taking the day when the Ap index crosses 20 as the epoch=0 time. For data set 2 (Supplementary Fig. 3), a large ensemble (500) of superposed epochs of 60 ‘pseudoevents’ selected randomly from the whole data set between 2002 and 2012 was created. For data set 3 (Supplementary Fig. 3 and Supplementary Table 2), ozone anomalies during winter, summer and spring/autumn are superposed on each other separately taking the day when the Ap index crosses 20 as the epoch=0 time. The analysis was carried out for each satellite and both hemispheres separately.

## Author contributions

M.E.A. and P.T.V. planned the research with input from A.S., C.J.R. and M.A.C. M.E.A. and P.T.V. carried out the data analysis and led the writing of the manuscript. C.J.R. and M.A.C. were responsible for processing the MEPED data and advised on statistical methods. A.S. provided expertise on GOMOS data and atmospheric dynamics. All authors contributed to the discussion and the writing of the final manuscript.

## Additional information

**How to cite this article**: Andersson, M. E. *et al.* Missing driver in the Sun–Earth connection from energetic electron precipitation impacts mesospheric ozone. *Nat. Commun.* 5:5197 doi: 10.1038/ncomms6197 (2014).

## Supplementary Material

Supplementary Figures and Tables Supplementary Figures 1-3 and Supplementary Tables 1-2

## References

[b1] Verronen P. T., Rodger C. J., Clilverd M. A. & Wang S. First evidence of mesospheric hydroxyl response to electron precipitation from the radiation belts. J. Geophys. Res. 116, D07307 (2011).

[b2] Andersson M. E. *et al.* Precipitating radiation belt electrons and enhancements of mesospheric hydroxyl during 2004-2009. J. Geophys. Res. 117, D09304 (2012).

[b3] Verronen P. T. *et al.* Comparison of modeled and observed effects of radiation belt electron precipitation on mesospheric hydroxyl and ozone. J. Geophys. Res. 118, 11419–11428 (2013).

[b4] Jackman C. H. *et al.* Northern hemisphere atmospheric effects due to the July 2000 solar proton events. Geophys. Res. Lett. 28, 2883–2886 (2001).

[b5] Seppälä A. *et al.* Destruction of the tertiary ozone maximum during a solar proton event. Geophys. Res. Lett. 33, L07804 (2006).

[b6] Brasseur G. P. & Solomon S. Aeronomy of the Middle Atmosphere 3rd edn. Springer (2005).

[b7] Rohen G. *et al.* Ozone depletion during the solar proton events of October/November 2003 as seen by SCIAMACHY. J. Geophys. Res. 110, A09S39 (2005).

[b8] Jackman C. H. *et al.* Short- and medium-term atmospheric constituent effects of very large solar proton events. Atmos. Chem. Phys. 8, 765–785 (2008).

[b9] Funke B. *et al.* Composition changes after the "Halloween" solar proton event: the High Energy Particle Precipitation in the Atmosphere (HEPPA) model versus MIPAS data intercomparison study. Atmos. Chem. Phys. 11, 9089–9139 (2011).

[b10] Langematz U. *et al.* Chemical effects in 11-year solar cycle simulations with the Freie Universität Berlin Climate Middle Atmosphere Model with online chemistry (FUB-CMAM-CHEM). Geophys. Res. Lett. 32, L13803 (2005).

[b11] Randall C. E. *et al.* NO_x_ descent in the Arctic middle atmosphere in early 2009. Geophys. Res. Lett. 36, L18811 (2009).

[b12] Seppälä A. *et al.* Arctic and Antarctic polar winter NO_*x*_ and energetic particle precipitation in 2002-2006. Geophys. Res. Lett. 34, L12810 (2007).

[b13] Rozanov E., Callisto M., Egorova T., Peter T. & Schmutz W. The influence of precipitating energetic particles on atmospheric chemistry and climate. Surv. Geophys. 33, 483–501 (2012).

[b14] Kodera K. & Kuroda Y. Dynamical response to the solar cycle: Winter stratopause and lower stratosphere. J. Geophys. Res. 107, 4749 (2002).

[b15] Lu H., Franzke C., Martius O., Jarvis M. J. & Phillips T. Solar wind dynamic pressure effect on planetary wave propagation and synoptic-scale rossby wave breaking. J. Geophys. Res. 118, 4476–4493 (2013).

[b16] Seppälä A., Lu H., Clilverd M. A. & Rodger C. J. Geomagnetic activity signatures in wintertime stratosphere wind, temperature, and wave response. J. Geophys. Res. 118, 2169–2183 (2013).

[b17] Rozanov E. *et al.* Atmospheric response to NO_*y*_ source due to energetic electron precipitation. Geophys. Res. Lett. 32, L14811 (2005).

[b18] Seppälä A., Randall C. E., Clilverd M. A., Rozanov E. & Rodger C. J. Geomagnetic activity and polar surface air temperature variability. J. Geophys. Res. 114, A10312 (2009).

[b19] Baumgaertner A. J. G., Seppälä A., Jöckel P. & Clilverd M. A. Geomagnetic activity related NO_*x*_ enhancements and polar surface air temperature variability in a chemistry climate model: modulation of the NAM index. Atmos. Chem. Phys. 11, 4521–4531 (2011).

[b20] Seppälä A. & Clilverd M. A. Energetic particle forcing of the northern hemisphere winter stratosphere: comparison to solar irradiance forcing. Front. Phys. 2, 25 (2014).

[b21] Jackman C. H., Roble R. G. & Fleming E. L. Mesospheric dynamical changes induced by the solar proton events in October-November 2003. Geophys. Res. Lett. 34, L04812 (2007).

[b22] Egorova T., Rozanov E., Zubov V., Schmutz W. & Peter T. Influence of solar 11-year variability on chemical composition of the stratosphere and mesosphere simulated with a chemistry-climate model. Adv. Space Res. 35, 451–457 (2005).

[b23] Schmidt H., Brasseur G. P. & Giorgetta M. A. Solar cycle signal in a general circulation and chemistry model with internally generated quasi-biennial oscillation. J. Geophys. Res. 115, D00114 (2010).

[b24] Merkel A. W. *et al.* The impact of solar spectral irradiance variability on middle atmospheric ozone. Geophys. Res. Lett. 38, L13802 (2011).

[b25] Kyrölä E. *et al.* GOMOS O_3_, NO_2_, and NO_3_ observations in 2002-2008. Atmos. Chem. Phys. 10, 7723–7738 (2010).

[b26] Rong P. P. *et al.* Validation of Thermosphere Ionosphere Mesosphere Energetics and Dynamics/Sounding of the Atmosphere using Broadband Emission Radiometry (TIMED/SABER) v1.07 ozone at 9.6*μ* in altitude range 15-70km. J. Geophys. Res. 114, D04306 (2009).

[b27] Smith A. K., López-Puertas M., García-Comas M. & Tukiainen S. SABER observations of mesospheric ozone during NH late winter 2002-2009. Geophys. Res. Lett. 36, L23804 (2009).

[b28] Waters J. W. *et al.* The Earth Observing System Microwave Limb Sounder (EOS MLS) on the Aura satellite. IEEE Trans. Geosci. Remote Sens. 44, 1075–1092 (2006).

[b29] Jiang Y. B. *et al.* Validation of aura microwave limb sounder ozone by ozonesonde and lidar measurements. J. Geophys. Res. 112, D24S34 (2007).

[b30] Evans D. S. & Greer M. S. NOAA Technical Memorandum Version 2.0 Space Environment Laboratory (2004).

[b31] Rodger C. J., Clilverd M. A., Green J. C. & Lam M. M. Use of POES SEM-2 observations to examine radiation belt dynamics and energetic electron precipitation into the atmosphere. J. Geophys. Res. 115, A04202 (2010).

