# Supplementary material for: Missing driver in the Sun–Earth connection from energetic electron precipitation impacts mesospheric ozone
Source: Nat Commun. 2014 Oct 14;5:5197. doi: 10.1038/ncomms6197 (PMC4214406; doi:10.1038/ncomms6197)
Supplement: Supplementary Figures and Tables — Supplementary Figures 1-3 and Supplementary Tables 1-2 [file ncomms6197-s1.pdf]

SUPPLEMENTARY INFORMATION

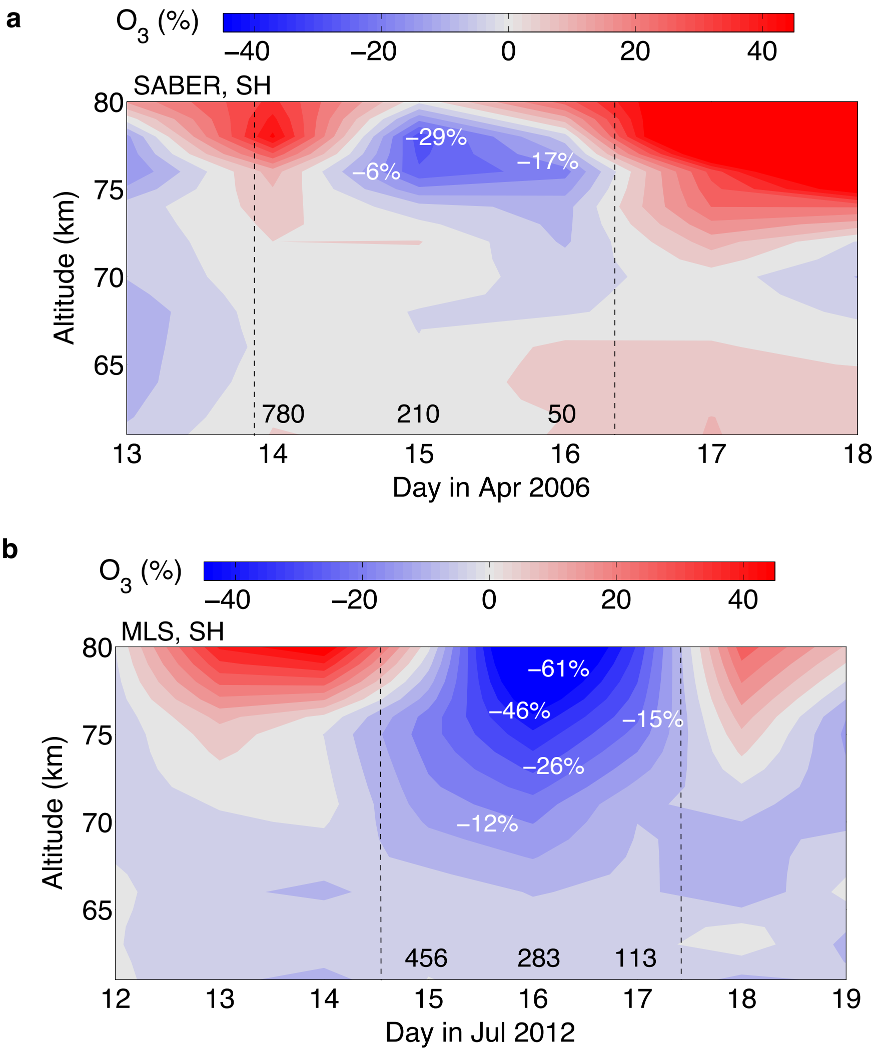

Supplementary Figure 1 Magnitude of the short-term EEP effects on mesospheric ozone. (a-b) O_3 anomalies (%) for two short EEP events (1-5 days) in the SH derived from SABER **(a)** and MLS **(b)** observations. Black dashed lines: EEP event start and end; black numbers: daily mean ECR; white numbers: O_3 loss (%) at different altitudes.

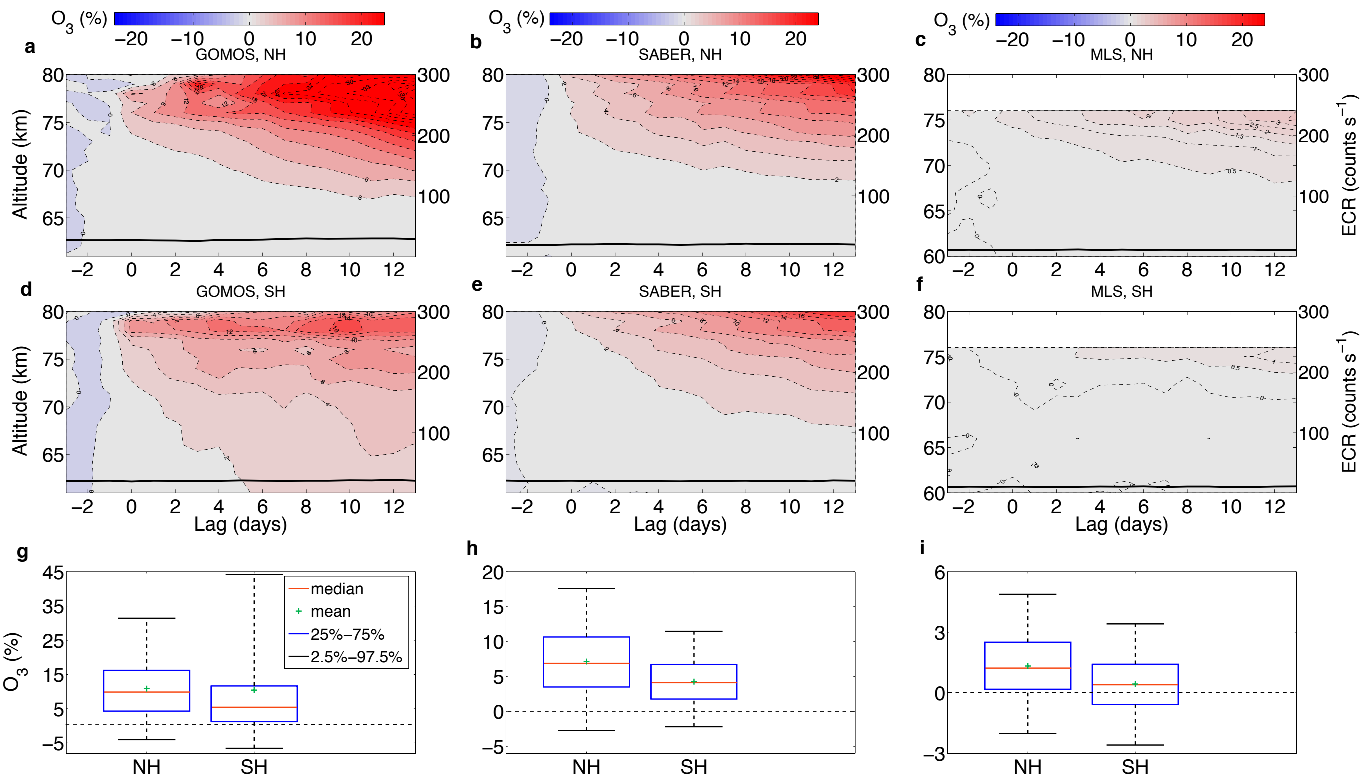

Supplementary Figure 2 Random Superposed Epoches. (a-f), Superposed epoch analysis for randomly selected cases (60 in total) showing ozone anomalies (%) in the NH **(a-c)** and in the SH **(d-f).** Ozone anomalies from GOMOS **(a, d)**. Ozone anomalies from SABER **(b, e)**. Ozone anomalies from MLS **(c, f)**. Black lines: mean electron count rates (ECR). The 95% and 50% confidence intervals, mean and median values for randomly selected epochs **(g-i)** calculated for the days between 0-8 and at altitudes with maximum ozone loss for each data set from 73 to 78 km (timing and altitudes taken from the EEP responses seen in Fig. 2).

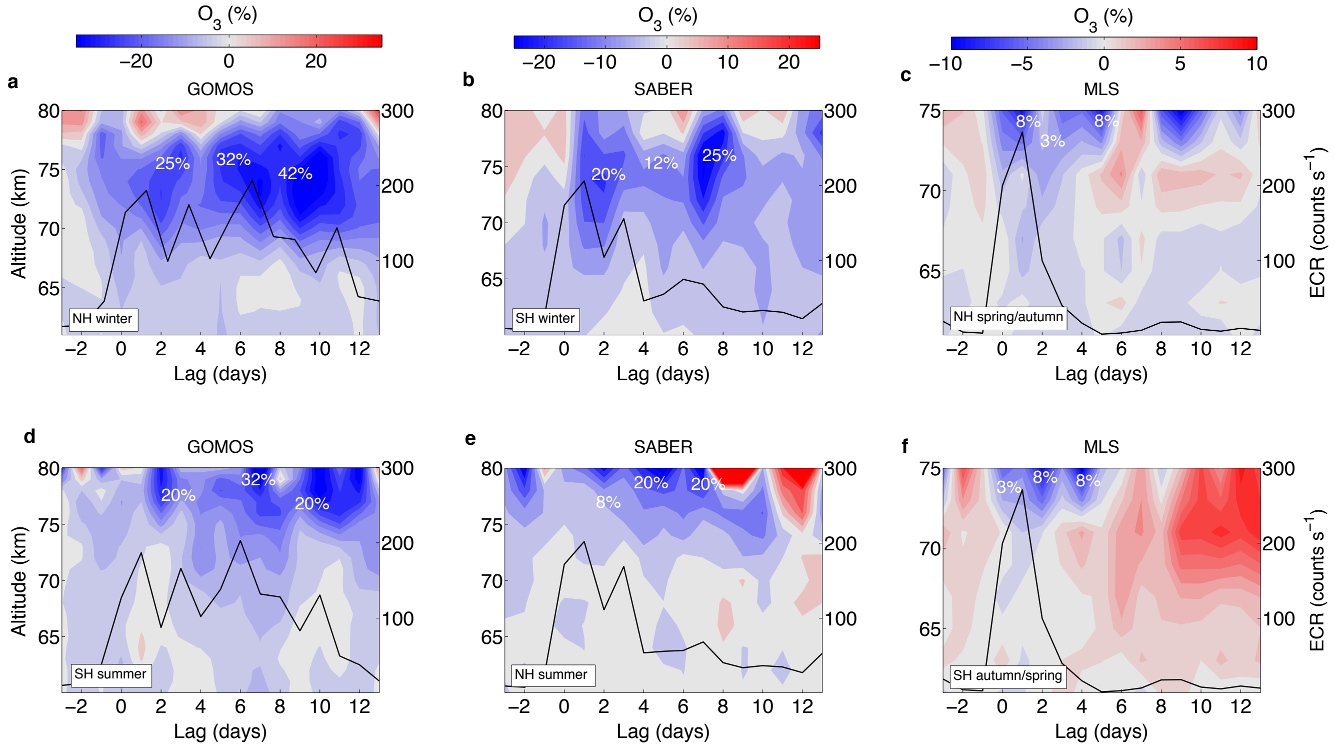

Supplementary Figure 3 Seasonal distribution. (a-f) Superposed epoch analysis for winter, summer and spring/autumn showing ozone anomalies (%). **(a, d)** NH winter and SH summer ozone anomalies (Nov-Feb) from GOMOS **(b, e)** SH winter and NH summer ozone anomalies (May-Aug) from SABER. **(c, f)** NH and SH spring/autumn ozone anomalies (Mar, Apr, Sep, Oct) from MLS. Black bars: mean electron count rates (ECR); white numbers: O_3 loss (%) at different altitudes.

Supplementary Table 1 Ozone data characteristics. Time=period of data availability. VR = approximate vertical resolution. SEM = mean value of the standard error of the daily mean. n_{min} and n_{max} are the typical minimum and maximum number of data profiles per day. The numbers are given for the 70-80 km altitude and separately for the geomagnetic latitudes 55-65° N/S.

Data	Time mm.yy	VR km	SEM (%) NH/SH	n_{min} NH/SH	n_{max} NH/SH
GOMOS	10.02-12.11	2	18/14	3/4	23/50
SABER	04.02-12.12	2	13/9	4/4	50/82
MLS	08.04-12.12	5	21/23	7/8	108/199

Supplementary Table 2 Seasonal distribution of EEP events between 2002-2012.

Data	Winter	Summer	Autumn/Spring
	NH/SH		
GOMOS	9/10	0/8	5/8
SABER	11/21	21/11	16/18
MLS	6/11	10/5	12/11
